# The Use of Mouse Models for Understanding the Biology of Down Syndrome and Aging

**DOI:** 10.1155/2012/717315

**Published:** 2012-02-23

**Authors:** Guido N. Vacano, Nathan Duval, David Patterson

**Affiliations:** Eleanor Roosevelt Institute, Department of Biological Sciences, The University of Denver, 2101 E. Wesley Avenue, Denver, CO 80208, USA

## Abstract

Down syndrome is a complex condition caused by trisomy of human chromosome 21. The biology of aging may be different in individuals with Down syndrome; this is not well understood in any organism. Because of its complexity, many aspects of Down syndrome must be studied either in humans or in animal models. Studies in humans are essential but are limited for ethical and practical reasons. Fortunately, genetically altered mice can serve as extremely useful models of Down syndrome, and progress in their production and analysis has been remarkable. Here, we describe various mouse models that have been used to study Down syndrome. We focus on segmental trisomies of mouse chromosome regions syntenic to human chromosome 21, mice in which individual genes have been introduced, or mice in which genes have been silenced by targeted mutagenesis. We selected a limited number of genes for which considerable evidence links them to aspects of Down syndrome, and about which much is known regarding their function. We focused on genes important for brain and cognitive function, and for the altered cancer spectrum seen in individuals with Down syndrome. We conclude with observations on the usefulness of mouse models and speculation on future directions.

## 1. Why Use Mouse Models?

Down syndrome (DS) is diagnosed by chromosome analysis, either prenatally (usually because of identified risk factors), or postnatally (typically because of the appearance of the infant). The DS phenotype is complicated and variable, thus models of DS must be able to address this complexity and variability.

Intellectual disability may be the most well-known feature of DS, but it is accompanied by behavioral, psychiatric, and neurological problems. In early infancy, people with DS function in the range of low typical development, but the intelligence quotient decreases in the first ten years of life, reaching a plateau in adolescence that extends into adulthood. Learning is complicated by a tendency to avoid cognitive challenges, and by a deficiency in language production. About 17.6% of individuals with DS less than 20 years of age have a psychiatric disorder, most often a disruptive behavioral disorder, such as attention deficit hyperactivity disorder, conduct/oppositional disorder, or aggressive behavior. About 25.6% of adults have a psychiatric disorder, most frequently depression or aggressive behavior. People with DS have a higher incidence of autism. By the fifth decade of life, neuropathological changes typical of Alzheimer's disease (AD) usually develop. Clinical signs and symptoms of AD are seen in 75% of people over 60 years of age. These are usually seizures, changes in personality, focal neurological signs, apathy, and loss of conversational skills [1].

The complexity of DS extends well beyond mental and neurological issues. For example, about half of people with DS are born with congenital heart disease, and heart disease can develop (or be initially identified) later in life. Adolescents and young adults with no known intracardiac disease can develop mitral valve prolapse and aortic regurgitation. People with DS are more likely to have hematological disorders. These include polycythaemia in newborns, macrocytosis, transient myeloproliferative disorder, acute myeloid leukemia, and acute lymphoblastic leukemia. Between 38% and 78% of people with DS have conductive and/or sensorineural hearing loss. About 38% of children less than 12 months of age, and 80% age 5 to 12, have ophthalmological disorders requiring monitoring and intervention. The most frequent disorders are refractive errors, strabismus, and nystagmus. Resting metabolic rates are reduced in individuals with Downs syndrome, which results in a higher frequency of obesity, and children at ages 3 to 4 are more likely to be obese than not. Monitoring intake of calcium and vitamin D is important, since individuals with DS exhibit lower bone density. People with DS have a higher incidence of coeliac disease and hypothyroidism. Many disorders, such as arthritis, atlantoaxial subluxation, diabetes mellitus, leukemia, obstructive sleep apnea, and seizures, occur more frequently among individuals with DS than in the general population [1].

Given the complexity of the DS phenotype, computer models, *in vitro* models, models based on lower organisms, and so forth, are woefully inadequate for representing DS. Mouse models have many characteristics that make them well suited to the study of DS. First, mice are a higher organism with the requisite biological characteristics. Neurological, behavioral, cardiac, hematological, skeletal disorders, and so forth, can be studied using mouse models. Second, they are very well characterized. Mouse models have been extensively used in research, and a great deal is known about them. Additionally, they are commonly used in development and testing of drugs for treatment of various disorders, including ones associated with DS. Third, there are numerous practical issues that make mouse models especially attractive. Mice are small, they have a relatively short generation time, they reproduce rapidly, are inexpensive to maintain and house, and are easy to handle.

## 2. What Defines a DS Mouse Model?

Various genetically altered mice have been proposed as mouse models of DS, and rapid progress is being made creating new models. An important point to bear in mind is that no mouse model will be a perfect model of DS. Even though mice have many similarities to humans, there are significant, and obvious, differences; therefore, some aspects of DS simply cannot be adequately modeled in mice. For example, it is clear that one can use mice to study aspects of learning and memory, but they cannot serve as a complete model for human intellect. Mice have their own sets of behaviors that have been selected for over evolutionary time, and some of these behaviors are not relevant to studies of humans, with or without DS. 

There are clear biochemical and metabolic differences between mice and humans as well, even though basic biochemical pathways have been conserved. For example, in humans, the end product of purine metabolism is uric acid, which is an antioxidant that may be relevant to the oxidative stress associated with DS. Indeed, individuals with DS accumulate unusually high levels of uric acid in their blood [2], which may be important in aging, and in neurodegenerative diseases associated with aging and DS [3]. This may be due to trisomy of the GART gene, which encodes an enzyme that catalyzes 3 steps of *de novo* purine synthesis, or it may be due to abnormal processing of uric acid by the kidneys of individuals with DS, or perhaps some other unknown mechanism. Mice, on the other hand, metabolize uric acid to allantoin, which is much more soluble and easily excreted. Therefore, modeling alterations in purine metabolism in mice may be difficult unless this metabolic difference is taken into account. One approach would be to use mice lacking uricase that accumulate uric acid as the end product of purine metabolism [4]. 

Another example is that mice metabolize folic acid somewhat differently than humans. Folate levels are about 10 fold higher in murine versus human plasma [5]. Therefore, alterations in folate metabolism in mice may not have the same metabolic consequences as similar alterations in humans. Abnormalities in folate metabolism or polymorphisms in genes encoding enzymes of folate metabolism have been associated with DS in many studies, although the significance of these polymorphisms is still unclear [6]. Adding to this complexity, it may be that particular polymorphisms in individual steps of folate metabolism may function only in the context of other polymorphisms, and that various suites of polymorphisms may have similar effects, making it difficult to compare studies [7]. Recent evidence also demonstrates that alterations in folate metabolism may be associated with specific aspects of DS, such as congenital heart disease [8]. Folate metabolism may also be related to the biology of aging and age-related disorders [9, 10]. Importantly, several genes necessary for folate metabolism reside on HSA21, including cystathionine beta synthase (CBS) [11]. Mutations in CBS are clearly associated with intellectual disability and cardiovascular disease [12]. The reduced folate carrier (Slc19a1), which is important for trafficking of folates in mammals, is also located on HSA21 [13]. Mutations or polymorphisms in Slc19a1 are associated with sensitivity to methotrexate [14]. It has been hypothesized that the presence of 3 copies of Slc19a1 in persons with DS may be partly responsible for their unusual sensitivity to folate analogues [15]. In the mouse genome, CBS maps to Mmu17 and Slc19a1 to Mmu10 (regions syntenic to HSA21). Numerous mouse models with alterations in CBS or Slc19a1 have been produced, including mice in which the endogenous mouse gene has been inactivated and replaced by the equivalent human gene [16–18]. So far, it has been difficult to learn much about human DS from studying these mice. The fact that these genes are present on different mouse chromosomes complicates the production of appropriate mouse models that are relevant to human DS.

Although Mmu16, 17, and 10 contain essentially all the known genes on HSA21, they are not completely analogous to HSA21. Some of the genes may be species specific in both humans and mice, and the chromosome regions may have regulatory sequences or copy number variations that may not encode proteins but are nonetheless important for phenotypic development. Sturgeon and Gardiner [19] have published an excellent comparison of the relevant mouse and human genetic regions (along with chimpanzee), and the interested reader is referred to that work for further details.

An additional consideration is that manipulation of the mouse genome may have unexpected consequences. As discussed below, some trisomy mouse models have recently been shown to have unexpected additional chromosomal alterations.

Nonetheless, several different types of mouse models have been extremely useful in investigating aspects of DS, and models are becoming more accurate and sophisticated. Therefore, analysis of various types of mouse models is likely to be increasingly important in unraveling specific aspects of DS, with different types of models having different roles.

It is important to carefully consider the usefulness of mouse models that clearly show phenotypes relevant to DS. Specifically, if a particular mouse model of DS has a phenotype(s) reminiscent of DS, is the model appropriate for investigating DS? As clinical trials based on studies of various mouse models become more common, this becomes an exceedingly important question (see Section 5). 

Even mice that do not show a phenotype reminiscent of DS may be quite useful in understanding DS. For example, mice in which a particular HSA21 syntenic gene has been inactivated by targeted mutagenesis may be crucial for understanding the function of that gene, informing understanding of its role in DS. Large-scale projects to inactivate every mouse gene individually and to evaluate the phenotype of each of the knockout mice are underway and should be extremely helpful in understanding the role of these genes in the DS phenotype (http://www.mousephenotype.org/). Moreover, these mice are useful in manipulating gene copy number in mice that are trisomic for particular Mmu chromosomal regions syntenic to HSA21. Comparison of several different models can increase confidence that phenotypes reminiscent of DS relate to human DS in a meaningful way. This approach was suggested in the first description of the isolation of the Ts65Dn mouse model, discussed below [20].

In this review, we have selected a few illustrative examples of mouse models of DS from the large number that exist. We describe mice trisomic for various regions of HSA21 or the mouse chromosomal regions syntenic to HSA21, selected transgenic (Tg) mice, and selected mice in which specific genes have been inactivated (knockout, or KO, mice). Further, where possible, we describe combinations of these models. We have focused primarily on genes for which both Tg and KO mice exist, and where KO mice have been combined with trisomic mice to elucidate function by restoring disomy. In this way, we have attempted to describe the wide range of options available in utilizing mouse models to study DS. We have also chosen genes that appear to be functionally related, for which considerable information regarding function is known, and that appear to be related to important DS phenotypes. Thus, we focus on the APP, RCAN1, and SYNJ1 genes because these appear to be important for synaptic plasticity and/or function, and are likely to be related to the intellectual disabilities seen in individuals with DS. We have also chosen RUNX1, ETS2, and ERG, a group of genes that are relevant to cancer. These genes are likely to be related to the altered incidence of cancer seen in individuals with DS. 

## 3. Types of DS Mouse Models 

### 3.1. Chromosomal Trisomy Mice

**Figure 1:**
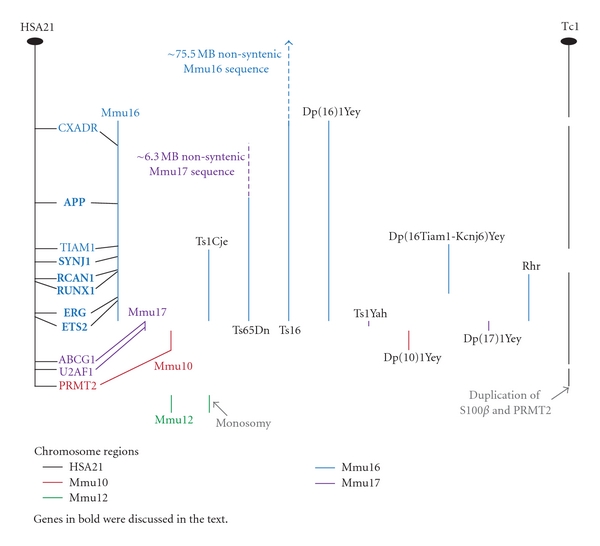
A graphical representation of HSA21 and the syntenic mouse chromosome regions from Mmu10, Mmu16, and Mmu17. The trisomic (or monosomic) chromosome regions present in 10 of the segmental mouse trisomies are also shown, with color-coding indicating the chromosome source of the region (see the key in the figure). The location of 11 HSA21 genes is shown, as well as their location on the syntenic chromosome regions, with text color indicating which syntenic chromosome. The dark ovals indicate the HSA21 centromere.

Mice trisomic for chromosome regions syntenic to HSA21, and mice that carry regions of HSA21, may be more complete models of the DS phenotype, since they are trisomic for many genes trisomic in individuals with DS. On the other hand, trisomy of multiple genes makes interpretation of results more complex. Numerous trisomic or transchromosomal mice have been produced. With the advent of chromosome engineering approaches, it is now possible to produce mice trisomic for any chromosome region.  Figure 1 shows a graphical representation of the chromosome regions present in these models, and a representation of HSA21 and the syntenic mouse chromosome regions.

#### 3.1.1. Ts16

The first mouse model of DS was the Ts16 mouse, which is trisomic for essentially all of Mmu16, including the part of Mmu16 syntenic to HSA21. These mice were produced by Alfred Gropp using a mouse breeding scheme that is selected for mice with centric fusions [21]. Several investigators, notably Dr. Charles Epstein, noted that some features of these mice were reminiscent of DS and hypothesized that these mice might present a model for certain aspects of DS [22]. This hypothesis was supported when several genes known to be located on HSA21, including SOD1, IFNAR, and GART, were found to be on Mmu16 [23–25]. This was the first evidence that mouse models of DS could be created. 

Unfortunately, the Ts16 mice generally die during fetal development or very shortly after birth. Thus, they have proven useful for the study of embryonic/fetal development but are not very helpful for studies of aspects of DS during the lifespan, and certainly not for aspects of DS relevant to aging. However, cell lines derived from Ts16 mice have been used to study biological processes related to DS and potentially relevant to aging and age-related disorders [26, 27]. An important caveat is that the Ts16 mice are trisomic for many Mmu16 genes that are not located on HSA21.

#### 3.1.2. Ts65Dn and Derivatives

The production by Muriel Davisson and colleagues of a mouse trisomic for only part of Mmu16, now known as the Ts65Dn mouse, was a seminal achievement in DS research [20]. They produced these mice by irradiating the testes of male mice, breeding them, and screening offspring for chromosomal rearrangements involving Mmu16. The Ts65Dn mouse is trisomic for roughly 94 genes syntenic to well-curated HSA21 genes, although this number is subject to change as analysis of the complete human and mouse genomic sequences continues [28]. It is the most well characterized and widely studied mouse model of DS. It is important to note that the Ts65Dn mouse is disomic for about 16 HSA21/Mmu16 genes [29], and it has recently been shown that these mice are also trisomic for a centromere proximal region of Mmu17. The chromosomal rearrangement site between Mmu16 and Mmu17 has been precisely defined [30, 31], and it turns out that Ts65Dn mice are trisomic for up to 60 Mmu17 genes, many of which are overexpressed in heart. Notably, two Mmu17 genes trisomic in Ts65Dn mice include Synj2 and Tiam2, which are related to the HSA21/Mmu16 encoded genes Synj1 and Tiam1. The phenotype of the Ts65Dn mouse is quite similar to the phenotype of mice trisomic for the entire region of Mmu16 syntenic to HSA21, and indeed to mice trisomic for all mouse chromosome regions present on HSA21 (discussed in more detail below). One difference between Ts65Dn mice and these models is that the trisomic region in Ts65Dn is present as a freely segregating extra chromosome, while in other models, the relevant chromosome region has been duplicated by chromosome engineering methods or in two cases (Ts1Cje and Ts2Cje) serendipitously. It has been argued that the presence of an extra chromosome in the Ts65Dn mice may make them a more acceptable model of DS. This proposal needs to be considered in light of observations on humans with DS due to translocations (i.e., not a freely segregating extra chromosome 21). The vast majority of these are Robertsonian translocations involving centromeric fusions [32]. Also, there are several examples of apparently balanced translocations between HSA21 and other human chromosomes that do not involve centromeres, resulting in an apparently classical DS phenotype [33–35]. Therefore, at least for phenotypes not related to reproduction, it seems that in humans the presence of an extra chromosome is not necessary for DS. 

Ts65Dn mice have many features reminiscent of those seen in people with DS. These include anatomical features such as small brain regions (notably the hippocampus and cerebellum) and abnormal skull shape [36]. Other similarities include congenital heart defects [37], myeloproliferative disorders [38], decreased bone density [39], and altered incidence and response to certain cancers [40, 41]. Most notably, Ts65Dn mice exhibit deficits in learning, memory and behavior, as well as aspects of early neurodegeneration that may be relevant to early features of AD as well as aging [42, 43]. 

Ts65Dn mice show signs of what might be called premature aging and neurodegeneration. They show early loss of basal forebrain cholinergic neurons that may be related to loss of learning and memory ability in these mice [42, 43]. Although the mice are trisomic for APP, they do not develop plaques or tangles characteristic of AD in humans or transgenic mouse models of this disorder. However, they do show increased expression of the APP gene and increased levels of the products of APP protein metabolism with age [44]. Moreover, old Ts65Dn mice accumulate tau/reelin containing clusters in the CA1 region of the hippocampus and extracellular tau/reelin granular deposits [45]. Similar deposits have been observed in mouse models of AD. Systemic aging has been examined in Ts65Dn mice, and there are indications that certain aspects of aging may be accelerated, for example, increased risk of lymphoma [46]. 

A number of treatments apparently improve learning and memory in Ts65Dn or prevent their decline [28]. Some of these are of particular relevance to the possibility of premature aging in these mice. For example, memantine, a drug used in humans to treat AD, appears to improve learning and memory in Ts65Dn [47–49]. Treatment of young (4 month old) Ts65Dn mice with a gamma secretase inhibitor has been reported to rescue learning and memory, potentially implicating the amyloid precursor protein (APP) or its metabolites in learning and memory deficits, even at an early age [50]. At least two laboratories have demonstrated that treatment with vitamin E can ameliorate learning and memory decline with age and concomitantly reduce signs of oxidative stress in the brains of Ts65Dn mice [51, 52]. One study suggests that vitamin E treatment is most useful if given perinatally and throughout life [52]. These findings are particularly relevant because numerous studies on the possible beneficial effects of vitamin E treatment on humans with AD and on humans with DS have been published or are underway. Although an early study appeared to show that large doses of vitamin E (2000 IU/day) slowed the loss of activities of daily living of persons with AD by about 11 months, other studies have not shown an effect, and the statistical significance of the initial study has been questioned [53–55]. A recent publication provides an interesting discussion of why human trials of vitamin E fail [56]. One obvious issue is that studies in which treatment has started after disease onset may fail because irreversible damage has already been done. This is consistent with studies suggesting that lifelong vitamin E supplementation of Ts65Dn mice may be most effective. 

A recent study also indicates that choline supplementation during pregnancy and lactation improves aspects of learning and memory and emotion regulation in adult Ts65Dn offspring [57]. This is consistent with earlier reports that prenatal choline supplementation improves the learning and memory of diploid rats, well into adulthood [58, 59]. More recent studies show that perinatal choline supplementation has beneficial effects on the development of the hippocampus in mice as well [60, 61]. These studies did not assess the long-term effects of perinatal choline supplementation on mouse behavior or learning and memory. This effect may be related to the cholinergic deficits seen in Ts65Dn mice.

Several other treatments, including fluoxetine, PTZ, prodrugs for norepinephrine, xamoterol, and perhaps lithium and voluntary exercise, improve certain aspects of the Ts65Dn phenotype. 

There have been a number of models derived from Ts65Dn mice. Some of these overcome specific weaknesses in the Ts65Dn model. For example, Ts65Dn mice carry a gene for retinal degeneration, which means some mice are blind and cannot be used for tests requiring vision. The gene has now been bred out of the Ts65Dn mice, and these mice appear otherwise to be essentially equivalent to the original Ts65Dn [62]. A second weakness of the Ts65Dn mouse is that males generally are functionally sterile. Ts2Cje mice are a Ts65Dn derivative in which the extra chromosome of the Ts65Dn mice has undergone a Robertsonian translocation with Mmu12 [63]. These mice appear to breed well, and males have increased, though still diminished, fertility. Recently, the Reeves laboratory has reported a method for breeding large numbers of Ts65Dn mice from Ts65Dn males [64].

#### 3.1.3. Ts1Cje

The Ts1Cje mouse is trisomic for a shorter region of Mmu16 than the Ts65Dn mouse, containing roughly 75 genes syntenic to well-curated genes located on HSA21. This mouse is the consequence of an attempt to inactivate Sod1 by targeted mutagenesis, resulting in a translocation between Mmu16 and Mmu12. The Sod1 gene is inactivated in these mice. It has recently been shown that this mouse is monosomic for seven Mmu12 genes [30, 65]. Two of these, Abcb5 and Itgb8, are related to the HSA21 genes ABCG1 (located on Mmu17), and CD18 (located on Mmu10). Neither of these is trisomic in Ts65Dn or Ts1Cje. 

The Ts1Cje mice are disomic for 19 genes that are trisomic in Ts65Dn (or Ts2Cje, which is apparently genetically equivalent, see Section 3.1.2) mice, including APP and SOD1. Therefore, a useful approach is to compare features associated with DS in the Ts2Cje and Ts1Cje mice. Presumably, differences between the two mouse strains are due to the difference in gene copy number, and common features are due to the common set of trisomic genes. An interesting comparison of Ts1Cje and the Ts2Cje mice indicates that both strains share enlarged brain ventricles and decreased neurogenesis [66]. On the other hand, learning deficits in Ts1Cje appear to be less severe than in Ts2Cje, and degeneration of basal forebrain cholinergic neurons is absent [67]. However, several neuroanatomical features related to DS, like regionally selective decrease in dendritic spines, are present in Ts1Cje but are less severe [68]. Given the recent recognition of the extent of trisomy of Mmu17 genes in Ts65Dn and the monosomy of Mmu12 genes in Ts1Cje, caution is necessary in interpreting the results of these comparisons. Moreover, as discussed above, it should be kept in mind that Ts65Dn mice are aneuploid and have a free extra chromosome, while Ts1Cje and Ts2Cje (and several of the mice described below) do not. The possibility exists that the presence of the extra chromosome in Ts65Dn mice may affect their phenotype [30]. 

An example of the usefulness of the Ts1Cje model has to do with the hypothesis that SOD1 and APP may be important for oxidative stress, mitochondrial dysfunction, and tau hyperphosphorylation, perhaps associated with premature aging and neurodegeneration. It appears that all these features are observed in the Ts1Cje mouse model even though APP and SOD1 are functionally diploid in these mice [69]. Presumably other genes trisomic (or possibly monosomic) in Ts1Cje play a role in these abnormalities.

Rapamycin, an inhibitor of mammalian target of rapamycin (mTOR), has recently been shown to extend the health span and lifespan of diploid, noninbred mice [70, 71]. mTOR is a key regulator of metabolism and of dendritic morphology and synaptic plasticity. Interestingly, in Ts1Cje mice, levels of BDNF and phosphorylated Akt-mTOR are elevated. This results in abnormally high local dendritic protein translation, thought to play a key role in memory formation. Treatment of Ts1Cje neurons with rapamycin repairs this defect [72]. These findings suggest the possibility that rapamycin, or other inhibitors of mTOR, might be useful in treatment of learning and memory loss and intellectual disability in DS. 

#### 3.1.4. Ts1Rhr and Related Mice

Mouse models have the potential to contribute to the question of genotype-phenotype mapping in DS. Appropriately constructed mouse models should be useful for testing whether the postulated Down Syndrome Critical Region (DSCR, a small region of HSA21 critical for the development of DS) exists [73, 74]. Olson et al. [75] used chromosome engineering to produce the Ts1Rhr mouse, which is trisomic for the mouse equivalent of the hypothetical human DSCR. This region includes about 33 genes. In their initial report, it was shown that this mouse DSCR is not sufficient and, by examining mice monosomic for this region, largely unnecessary for the craniofacial phenotype seen in Ts65Dn mice and in people with DS. In later studies [76], it was shown that trisomy of the DSCR alone is necessary, but not sufficient, for the structural and learning and memory deficits (assessed by the Morris water maze) seen in Ts65Dn mice and in DS. However, a later, more comprehensive study utilizing behavioral tests considered more sensitive than the Morris water maze test revealed that the situation is considerably more complex than initially thought [77]. In this study, using the same mice, trisomy of this region was found sufficient to confer behavioral, neurophysiological, and synaptic phenotypes characteristic of DS. In all, 20 of 48 features related to DS were altered; however, some changes were less severe than in Ts65Dn (or Ts1Cje) mice. Moreover, the Ts1Rhr mice showed phenotypes that are not observed in Ts65Dn or other models, including increased body and brain weight and a larger posterior hippocampal region compared to diploid mice, which is not seen in DS and in Ts65Dn or Ts1Cje mice. The authors suggest that these findings may mean that people with partial trisomy 21 may have phenotypes not seen in full trisomy 21. As mentioned above, Ts65Dn mice have reduced bone density [39]. Ts1Rhr mice do not exhibit this phenotype, and mice monosomic for this region show decreased bone density [78]. These experiments illustrate the complexity of the DS phenotype and reinforce the concept that study of different mouse models is important for developing an understanding of how DS develops. 

#### 3.1.5. Ts1Yah

Ts1Yah mice are trisomic for the HSA21 syntenic region on Mmu17 between Abcg1 and U2af1, which contains 12 genes [79]. These mice have several interesting features. They have learning and memory deficits as measured by the open field, Y arm maze, and novel object recognition tests. However, they appear to learn more efficiently in the Morris water maze test than diploid mice. They also have larger and longer lasting long-term potentiation (LTP) responses than diploid control mice, probably related to their improved performance on the Morris water maze. This is a provocative finding that clearly supports the hypothesis that interaction of many regions of HSA21 is required for the DS phenotype. Indeed, as the authors point out, trisomy of certain genes or regions of HSA21 may actually improve some aspects of cognition. Such a compensatory mechanism has been hypothesized to exist in human DS as well [80].

Ts1Yah mice also illuminate some of the necessary precautions required when studying chromosomally engineered mice. Expression levels of the genes within the trisomic region (and in the companion monosomic mice) were measured. Expression of the two genes at the end of the triplicated or deleted region, ABCG1 and U2af1, was not altered in monosomic, disomic, or trisomic mice. Ubash3a, Tff2, Tff3, and Tmprss3 were expressed equally in monosomic and trisomic regions. The other genes in the region were expressed according to gene dosage. The Umodl1 gene, adjacent to the Abcg1 gene but not in the engineered region, showed apparent increased expression in the thalamus, but not in the hippocampus and cerebellum. These results demonstrate the importance of analyzing chromosomally engineered mice for unexpected effects on expression of genes near the rearranged chromosomal region.

#### 3.1.6. Mice Trisomic (or Monosomic) for the Entire Mmu16, 17, and 10 Chromosomal Regions Syntenic to HSA21

It could be argued that the ideal mouse model of DS would be trisomic for all the mouse genes syntenic to HSA21 (i.e., the relevant parts of Mmu16, 17, and 10). A seminal achievement has been accomplished via a process involving production of mice trisomic for the relevant regions of each mouse chromosome using chromosomal engineering methods [29]. Then, mice trisomic for all three regions were produced via selective breeding. These mice have many features reminiscent of those seen in DS. Many of the abnormalities in learning, memory and hippocampus are very similar to those seen in the Ts65Dn mice. 

The production of these mice allows examination of the effect of trisomy of each syntenic chromosome region individually [81], resulting in some intriguing findings. Dp(10)1Yey/+ mice, trisomic for the Mmu10 syntenic region, did not have any detected alterations in learning and memory behaviors or in hippocampal LTP. Trisomy of the syntenic region on Mmu17 in Dp(17)1Yey/+ mice resulted in an increase in hippocampal LTP but no statistically significant change in learning and memory as assessed by the Morris water maze or contextual fear conditioning. The Dp(16)1Yey/+ mice (trisomic for the Mmu16 region syntenic to HSA21), on the other hand, showed abnormalities in hippocampal LTP and both the Morris water maze and contextual fear conditioning tests similar to those seen in the Ts65Dn mice. These results allow for some preliminary conclusions regarding the Ts65Dn mice and demonstrate the value of multiple mouse models. Specifically, since the Dp(16)1Yey/+ and the Ts65Dn mice have similar phenotypes, one could argue that the extra Mmu16 genes trisomic in the Dp(16)1Yey/+ mice do not contribute to these aspects of the Ts65Dn phenotype. Also, since the Dp(16)1Yey/+ mice are not trisomic for any of the Mmu17 genes trisomic in Ts65Dn, these genes are also unlikely to be important for the measured phenotypic changes. Finally, the minimal effect of the Mmu10 and 17 genes on the measured phenotypes suggests that these genes are not major contributors to the DS related phenotypes. However, these studies do provide evidence that the various chromosome regions may interact with each other, so care must be taken in making these conclusions. One must also keep in mind that variations in the tests used may lead to different interpretations regarding the relationship of the phenotype of these mice to DS. Also, more complete characterization of these mice may yet reveal that trisomy of the Mmu17 or 10 regions does lead to phenotypic alterations relevant to DS.

One important phenotype of DS is congenital heart defects. Considerable effort has been spent attempting to correlate partial trisomies of HSA21 with this phenotype [73, 82]. Analysis of the mice described above indicates that only the Mmu16 region is required to produce heart defects in mice. In an elegant extension of this work, the region associated with heart defects has been further delineated. Mice carrying either a 5.43 Mb duplication or the corresponding deletion of a region extending from and including the *Tiam1* and *Kcnj6* genes, Dp(16Tiam1-Kcnj6)Yey/+ and Df(16Tiam1-Kcnj6)Yey/+, were produced by chromosomal engineering [83]. These experiments, including breeding the Dp(16)1Yey/+ mice with the Df(16Tiam1-Kcnj6)Yey/+ to restore disomy of the genes in this region, demonstrate that trisomy of the Tiam1-Kcnj6 region is necessary and sufficient to produce heart defects in mice. This approach is logically similar to the approach of breeding trisomic mice with knockouts of individual genes to assess the role of these genes in various DS phenotypes, described below. 

#### 3.1.7. Tc1 (Human Transchromosomal)

A caveat of trisomic mouse models is the possibility that increased dosage (“trisomy”) of HSA21 genes may produce different phenotypic effects. Some investigators have argued that a mouse in which HSA21 has been stably introduced into the mouse genome would be a better model of DS. In 2005, O'Doherty et al. [84] reported the production of a mouse carrying an HSA21 that was missing a small number of HSA21 genes. This HSA21 was reported to have about 91% of the full complement of HSA21 genes. The mouse has many features seen in individuals with DS. However, so far, all Tc1 mice are mosaics. That is, the chromosome is present in a variable number of cells in any tissue. More recently, it has been reported that the HSA21 in the Tc1 mouse only contains 81% of the full complement of HSA21 genes [85]. In addition, this chromosome apparently contains a duplication of the S100*β* and PRMT2 genes [30]. Tc1 mice lose the extra HSA21 chromosome on an inbred pure genetic background. Moreover, some of the phenotypes of these mice depend upon the genetic background of the animals. This is not a surprising result and provides another cautionary note regarding the use of mouse models. 

### 3.2. Transgenic Mice

Transgenic mice contain additional, artificially introduced foreign genetic material, often a single gene, resulting in gain of function or overexpression of a certain protein(s). The use of transgenic mice provides an opportunity to study the biochemical and phenotypic implications of overexpression of individual trisomic genes *in vivo*. Molecular cloning of individual HSA21 encoded genes allows analysis of their expression and organization of their products and possible contributions to the DS phenotype. Features of transgenic mice that should be considered include gene copy number, levels of transcription and protein expression, tissue specificity and timing of expression, the site of integration of the transgene, and the genetic background of the mice. Figure 1 shows the location of these genes (indicated in bold) on HSA21 and the syntenic mouse chromosome regions.

### 3.3. Transgenic Mice Possibly Related to Intellectual Disability and Altered Brain Function in Individuals with DS

Several genes on HSA21 have been found to be important for neurodegenerative disorders, notably AD and amyotrophic lateral sclerosis (ALS, or Lou Gehrig's disease) or for synaptic function and neurological development and degeneration. 

#### 3.3.1. APP

APP (aka AAA, AD1, PN2, ABPP, APPI, CVAP, ABETA, PN-II, and CTF*γ*; App is the murine homolog) encodes the amyloid beta (A4) precursor protein, which is a cell surface receptor and transmembrane precursor protein. Multiple transcript variants encoding different isoforms have been found for this gene. It is overexpressed in some trisomic mouse models and in individuals with DS [86, 87]. APP is concentrated at the synapse in neurons and may play a role in synapse formation and plasticity [88–90]. Typically, APP undergoes extensive posttranslational processing including phosphorylation, glycosylation, and proteolysis. Normal APP proteolysis involves cleavage of the extracellular domain by an *α*-secretase followed by cleavage of the intermembrane domains by *γ*-secretase. The amyloidogenic pathway caused by abnormal cleavage of APP by *β*-secretase leads to aggregation of beta-amyloid peptide after cleavage by *γ*-secretase. The production of amyloid plaques is considered a hallmark neuropathological feature of AD. The protein is cleaved by secretases producing a number of peptides. Some of the peptides are secreted and bind to the acetyltransferase complex APBB1/TIP60 to promote transcriptional activation. Other peptides are components of the amyloid plaques found in the brains of patients with AD. Mutations in this gene have been implicated in autosomal dominant AD and cerebroarterial amyloidosis. Early in life, individuals with DS begin to develop progressive aggregation of beta-amyloid peptide and AD-like neuroanatomical features.

Initially, transgenic models overexpressing wild-type (WT) APP did not result in development of a neurodegenerative condition or AD-like pathologies such as amyloid plaques. Though there are several WT APP transgenic lines, only one appears to form plaques [91].

Using mice transgenic for WT human APP, Salehi et al. [92] demonstrate that increased APP expression results in a modest but significant decrease in nerve growth factor (NGF) transport. 

Simón et al. [93] show that overexpression of WT APP in mice results in multiple pathological features, including cognitive deficits, severe histopathological abnormalities in cytoskeleton, and signs of synaptic dysfunction, as well as evidence of cell loss in the hippocampus and entorhinal cortex. These alterations are accompanied by an early increase in phosphorylated tau protein and elevated levels of APP derived carboxy-terminal fragments but, remarkably, almost undetectable levels of A*β* peptide. These results strongly suggest the presence of A*β* independent pathogenic pathways in AD.

The discovery of familial AD (FAD) mutations led to the overexpression of mutant APP in transgenic mice that does induce plaque pathology. These mice recreate many of the pathologies associated with AD, including early-onset AD as seen in DS. Overexpression of WT APP in DS is associated with early onset AD [94]. Recently, it was found that overexpression caused by APP gene duplication might lead to FAD [95–97]. 

Overexpression of APP in these models is often at levels far exceeding physiological levels, often up to 10-fold higher. It has been suggested that overexpression of APP, or any protein for that matter, at such high levels may be toxic. In humans, amyloidopathy often results in a progressive neurodegenerative condition; in mice this seldom is the case. Though amyloidopathy does cause cognitive decline, it is more reminiscent of natural aging or a predementia stage rather than a complete neurodegenerative disease (reviewed in [98]). In fact, the level of plaque load does not correlate well with severity of cognitive decline in people with AD [99]. 

The use of transgenic APP mouse models as models of AD as well as models of aging is further discussed [98, 100, 101]. For a general list of transgenic APP, as well as other models for neurodegenerative disease, the interested reader is referred to the Alzheimer's forum: http://www.alzforum.org/res/com/tra/.

#### 3.3.2. RCAN1

The regulator of calcineurin 1 gene (RCAN1, aka CSP1, DSC1, RCN1, DSCR1, MCIP1, ADAPT78; Rcan1 is the murine homolog) encodes the calcipressin-1 protein, which interacts with calcineurin A, and inhibits calcineurin-dependent signaling pathways (such as activation of nuclear factor of activated T-Cells (NFAT) transcription factors) [102]. The gene is overexpressed in brain of DS fetuses. RCAN1 is up regulated by calcineurin signaling, suggesting regulation via a negative feedback loop. Calcineurin is an ubiquitously expressed Ca^2+^-dependent phosphatase abundant in both the developing and adult brain, heart, skeletal muscle, and endocrine tissue [102–104]. It is responsible for many Ca^2+^-dependent neuronal functions including neurotransmitter release, neurite outgrowth, cytoskeletal stabilization, and apoptosis (reviewed in [105]). 

In trisomic mice, such as the Ts65Dn model, and in DS fetal tissue, RCAN1 is increased by up to 1.8 fold possibly affecting CNS development [40, 104]. In individuals with sporadic AD, RCAN1 is overexpressed in the cerebral cortex and hippocampus, and chronic overexpression may lead to neurofibrillary tangles associated with AD pathology [104]. Ca^2+^ induces the expression of RCAN1 in a calcineurin-dependent manner creating a negative feedback mechanism causing sustained calcineurin repression [106]. Therefore, the regulation of calcineurin by RCAN1 is of significant importance in the pathology of DS and AD.

Transgenic mice generated using a human RCAN1 cDNA splice variant 1 under the control of the endogenous promoter show a 4-fold increase in expression. Chromaffin cells taken from the transgenic mice show disruption in exocytosis and vesicle trafficking mechanisms in a non-calcineurin-dependent manner [107]. The authors make apparent that these results are from a transgenic model with a 4-fold increase in expression, which is much higher than the 1.5–2 fold increase typically found in individuals with DS. 

 A similar RCAN1 transgenic mouse was generated under the control of the platelet-derived growth factor beta (PDGF*β*) promoter to drive expression in the brain [108]. These mice show a 1.3–1.5 fold overexpression in the hippocampus and cerebral cortex along with poor performance in the Morris water maze, indicating a disruption in visuospatial learning. However, no differences in performance of memory tasks were observed, suggesting once a task was learned, retention was not impaired [108]. The authors conclude that RCAN1 overexpression may contribute to a disruption in the calcineurin-dependent phosphorylation/dephosphorylation balance in the hippocampus and may inhibit learning, but not memory. 

#### 3.3.3. SYNJ1

Phosphatidylinositol-4, 5-bisphosphate is an important intracellular signaling phospholipid and plays essential roles in signal transduction, membrane trafficking, and cytoskeletal dynamics [109–111]. Because it plays a significant role in several cellular signaling events, balance at the cellular membrane is crucial. Phosphate kinase type-1*γ* (PIPK1) and synaptojanin 1 (SYNJ1) are critical to maintaining this balance at neuronal synapses [112, 113]. Synaptojanin 1 may act by dephosphorylating PtdIns(4,5)P_2_ and may help stabilize PIPK1 [111, 113]. 

SYNJ1 is found on HSA21 and is trisomic in individuals with DS [87, 114]. Considering the vital role SYNJ1 plays in cellular dynamics through PtdIns(4,5)P_2_ regulation, dysfunction of PtdIns(4,5)P_2_ metabolism through SYNJ1 overexpression may result in neurophysiological changes seen in DS and may contribute to early onset AD pathology [115, 116]. Individuals with DS develop the pathology of AD by their 3rd decade, possibly due to the overexpression of APP and an increase in beta amyloid plaques [117]. Additionally, the overexpression of SYNJ1 due to trisomy of HSA21 may render neurons more sensitive to the insults of beta amyloid [116]. 

Transgenic mice were generated using BAC constructs for both human and mouse SYNJ1 genes [115]. The human and mouse SYNJ1 transgenic mice presented a 2.5-fold increase in transcript levels and a 59% and 38% increase in protein levels. Overexpression of SYNJ1 resulted in altered PtdIns(4,5)P_2_ metabolism in the brains of these mice. These authors suggest that given the pleiotropic nature of PtdIns(4,5)P_2_, irregularities in the metabolism of PtdIns(4,5)P_2_ could have significant effects on many different cellular functions. In addition to the altered PtdIns(4,5)P_2_ metabolism, these mice exhibit poor performance in the Morris water maze suggesting deficits in cognition and learning [115].

### 3.4. Transgenic Mice Possibly Related to the Altered Cancer Spectrum in People with DS

Individuals with DS have an altered spectrum of cancers. Specifically, there is a significantly increased risk of childhood leukemia and a significantly decreased risk of some solid tumors including many for which incidence is age related [118]. Some trisomic mice have similar features. Therefore, considerable work has been done with transgenic and KO mice with altered levels of genes encoded on HSA21 thought to be relevant to cancer. 

#### 3.4.1. ETS2

The v-ets erythroblastosis virus E26 oncogene homolog 2 (avian) (ETS2, aka ETS2IT1; Ets2 is the murine homolog) encodes protein C-ets-2, a transcription factor, and is a prototype of the ETS family of transcription factors. The gene for ETS2 is found on HSA21. The ETS family of transcription factors activate or repress genes responsible for cellular proliferation, differentiation, stem cell development, cellular transformation and tumorigenesis, cell senescence, and apoptosis [119]. The conserved ETS domain within these proteins is a winged helix-turn-helix DNA-binding domain that binds the core consensus DNA sequence GGAA/T of target genes [120]. Ets2 is essential for trophoblast development and is involved in establishing the AP axis and paraxial mesoderm during development. 

Overexpression of Ets2 has been shown to increase apoptosis and is linked to DS pathophysiology [121–123]. Ets2 transcription factors are found in neurons and seem to be critical for neuromuscular junction formation in mice [124]. Mouse models with less than a 2-fold overexpression of Ets2 show neurocranial, viscerocranial, and cervical skeletal abnormalities reminiscent of trisomy 16 mouse models and individuals with DS [125]. This model expresses the Ets2 cDNA transgene under the control of a metallothionein promoter causing ubiquitous overexpression. These phenotypes are reminiscent of physiological conditions of individuals with DS and trisomic mice [125].

Using ETS2 transgenic mice, Wolvetang et al. [126] show that the Ets2 transcription factors activate the APP gene via specific Ets binding sites, acting cooperatively with the AP1 transcription factor. Furthermore, brains and primary neuronal cultures from ETS2 transgenic mice and from fibroblasts overexpressing ETS2 display abnormalities reminiscent of DS such as elevated APP protein and beta-amyloid production [126]. This may exacerbate the effects adverse effects caused by APP overexpression in individuals with DS. 

#### 3.4.2. RUNX1

The runt-related transcription factor 1 gene (RUNX1, aka AML1, CBFA2, EVI-1, AMLCR1, PEBP2aB, and AML1-EVI-1; Runx1 is the murine homolog) encodes runt-related transcription factor 1. RUNX1 is a hematopoietic transcription factor associated with normal hematopoiesis and megakaryopoeisis development [127, 128]. RUNX1 protein forms a heterodimeric transcription complex with core-binding factor *β* (CBF*β*). This complex is the most common target observed in leukemia-associated translocations, suggesting that it has an important role in regulation of normal hematopoiesis. Children with DS are more likely to develop leukemia, and 10% of children with DS are born with transient megakaryoblastic leukemia (TML), which often develops into acute megakaryocytic leukemia (AMKL) [129]. RUNX1 is responsible for the terminal differentiation of megakaryocytic progenitors. Mutations, and translocations of RUNX1 are associated with acute myeloid leukemias (AMLs) [128]. However, trisomy of RUNX1 does not seem to be directly involved in TML or the progression of AMKL in DS [130–132]. 

Transgenic mice expressing mouse Runx1 under the control of the GATA1 hematopoietic regulatory domain (HRD) were generated to determine the role of Runx1 in the development myeloid leukemia in mice [133]. These mice show roughly a five-fold overexpression of Runx1 transcript and protein in whole bone marrow. It was determined that a 5-fold increase in Runx1 did not initiate an increase in leukemia. However, this group proceeded to cross the transgenic Runx1 mouse with the BXH2 mouse model of myeloid leukemia effectively adding an additional copy of Runx1. These mice show a decrease in the time period of myeloid leukemia onset [133]. The overexpression of Runx1 in the Runx1-BHX2 cross is reminiscent of childhood DS, AMKL, and similar to children with DS, this condition was preceded by TML. However, these mice show a 5-fold increase in Runx1 expression levels initiating TML and AMKL, which to date has not been reported in children with DS. 

Interestingly, RUNX1 physically interacts with GATA1 [134]. GATA1 has been shown to be dysfunctional in children with DS and in the development of AMKL [135–138]. It has been suggested that an overdose of RUNX1 may render GATA1 dysfunctional, and this may lead to the development of AMKL in children with DS [139].

### 3.5. Mice with Genes Inactivated by Targeted Mutagenesis

Gene deletion is a powerful method for investigating gene function, and for determining whether or not a gene is essential for viability. It is also useful for evaluating genes via manipulation of gene dosage in the context of two separate hypotheses; the “gene dosage effect,” in which abnormal expression of individual genes is responsible for specific DS features, and “developmental instability,” in which homeostasis is disrupted by chromosomal imbalance and aberrant expression of many genes, resulting in developmental abnormalities. 

### 3.6. Knockout Mice Possibly Related to Intellectual Disability and Altered Brain Function in Individuals with DS

#### 3.6.1. APP

An App null mutant was generated via gene targeting using a vector designed to replace the App promoter, exon 1, and part of the first intron with a neomycin phosphotransferase gene (PGKneo) cassette [140]. Neither App mRNA nor protein was detectable in App null animals, however, these mice are viable and do not display any overt abnormalities. Neuroanatomical analysis of brain tissue did not show any significant differences versus WT. However, Heber et al. [141] demonstrated that the App functions are indeed essential. App is one member of a gene family including amyloid beta (A4) precursor-like protein 1 and 2 genes (Aplp1 and Aplp2). They demonstrate that mice null for Aplp2 have no apparent abnormalities, but mice null for both App and Aplp2 exhibit perinatal lethality, indicating redundancy. They obtained similar results with mice null for both Aplp1 and Aplp2, which suggests a critical role for Aplp2. Mice null for both App and Aplp1 are viable. Surprisingly, mice null for both App and Aplp2 show no obvious histopathological abnormalities in brain, and cortical neurons showed normal survival in basal culture.

Salehi et al. [92] demonstrated that, in Ts65Dn, there is a marked decrease in nerve growth factor (NGF) transport in hippocampus, resulting in down regulation of nerve growth factor receptor (NGFR, or p75NTR) gene expression and deterioration of basal forebrain cholinergic neurons (BCFN). In Ts1Cje mice, there is a very mild decrease, when compared to euploid mice. The authors hypothesize that the marked decrease is due to trisomy of the App gene, which is trisomic in Ts65Dn, but not in Ts1Cje. Crossing Ts65Dn with a null allele for App (i.e., bringing the App gene dosage from trisomy to disomy) partially rescues the NGF decrease. Consistent with this observation, mice transgenic for a human APP allele, which expresses the gene at levels comparable to levels in DS, show a (relatively mild) reduction in NGF transport. 

These observations suggest that trisomy of App is largely, but not exclusively, responsible for the decrease in NGF transport and the resulting reduction in BCFN seen in Ts65Dn. Mice transgenic for both APP and presenilin 1 (PSEN1, or PS1) show further reduction in NGF transport and in BCFN number, indicating an additive effect, further supporting this hypothesis.

Early endosomal alterations are the earliest known pathology in sporadic AD and DS [142]. These alterations appear before birth in DS, and in AD, prior to the deposition of *β*-amyloid and as soluble A*β* levels first rise. The alterations have been observed in the hippocampus, neocortex, and basal forebrain.

The endosomal alterations are seen in Ts65Dn mice, but are not seen in Ts1Cje mice (which are disomic for App), or in Ts65Dn disomic for App (Ts65Dn, App+/+/−), which indicates that increased App expression is required for the alterations. However, the alterations are not present in mice transgenic for either the human APP London (APP670/671 plus APP717) or Swedish (APP670/671) mutations. Both mutations result in high expression of APP (two fold for the London transgenic, and seven fold for the Swedish transgenic). These results indicate, collectively, that App overexpression is necessary, but not sufficient for producing the alterations; overexpression of one or more additional MMU16 genes trisomic in Ts65Dn is required. The endosomal alterations may be at least partially due to reduction in NGF transport [143].

#### 3.6.2. RCAN1

An Rcan1 null allele was generated by gene targeting using a vector designed to replace exons 5 and 6 with *β*-galactosidase [144]. Null (−/−) Rcan1 mice are viable and fertile, and exhibit no overt abnormalities. Northern blot analysis demonstrates that the null allele does not produce detectable transcript.

Calcineurin has been shown to be necessary and sufficient for cardiac hypertrophy, in response to various physiological and pathological stimuli. KO mice lacking the calcineurin A *β* catalytic subunit exhibit diminished response to hypertrophic stimuli. Since calcipressin-1 inhibits calcineurin-dependent signaling, increased expression of calcipressin-1 would be expected to reduce the hypertrophy response, and decreased expression should increase hypertrophy. Consistent with this expectation, Rcan1 null mice carrying a muscle-specific transgene expressing activated calcineurin showed an exacerbated hypertrophic response, and severe fibrosis. Unexpectedly, cardiac hypertrophy was reduced in null mice in which the hypertrophic stimulus was due to aortic banding, or chronic adrenergic stimulation. This suggests that calipressin-1 may have a dual role in cardiac hypertrophy, dependent on differences in hypertrophic stimulation.

Rcan1 is expressed in mouse in developing brain and craniofacial structures. It is trisomic in several DS mouse models, including Ts65Dn, Ts1Cje, and Ts16. Ts16 embryos exhibit high incidence of cardiac valvuloseptal malformations and abnormal development of the brain, skull, and sensory organs. However, mice trisomic for a Mmu16 region syntenic to an HSA21 region (Ts1Rhr [75]), but not including the Rcan1 locus (among other loci), do not develop cranial dysmorphologies, suggesting the possibility that overexpression of Rcan1 may be partially or fully responsible for these phenotypes.

Lange et al. [145] evaluated expression of Rcan1 in the Ts16 mouse model and show that expression of Rcan1 isoforms is increased in developing heart and brain, versus diploid littermates, while NFAT transcriptional activity is decreased. To evaluate the role of Rcan1 in Ts16 trisomy, they employed a breeding strategy using the Rcan1 null mice [144] to restore the Rcan1 locus to disomy, in the Ts16 background. Examination of these mice demonstrates that restoring Rcan1 to disomy in Ts16 mice does not rescue cardiac and craniofacial abnormalities.

It has long been known that the incidence of many cancer types (typically solid tumors) is reduced in individuals with DS, and this protection is thought to be due to increased expression of one or more of the chromosome 21 genes that are trisomic in DS. RCAN1 suppresses vascular endothelial growth factor- (VEGF-) mediated angiogenic signaling via the calcineurin pathway. Baek et al. [40] demonstrated that RCAN1 is expressed about 1.8 fold higher in DS fetal tissues, and Rcan1 is expressed about 1.7 fold higher in Ts65Dn mice. They tested two tumor models, Lewis lung carcinoma and B16F10 melanoma, in Ts65Dn mice, and observed considerable tumor growth suppression relative to WT, accompanied by a decrease in microvessel density. They obtained similar results using an Rcan1 transgenic mouse, and also noted a significant decrease in CD31^+^CD45^−^ cells (CD31 is an endothelial marker, CD45 is a hematopoietic marker) versus WT. Inoculation of transgenic and WT mice with reduced numbers of Lewis lung carcinoma cells to generate slowly growing tumors demonstrated that increased Rcan1 expression inhibits the initial expansion as well as extended growth of transplanted tumors, indicating inhibition of both neoangiogenesis and co-option of existing blood vessels.

Matings were performed to produce Ts65Dn/Rcan1^+/+/−^ mice, which exhibit significantly abrogated tumor protection along with increased microvessel density, demonstrating that Rcan1 overexpression plays an important role in these processes [40]. Since increased Rcan1 dosage attenuates VEGF-calcineurin-NFAT signaling, the authors examined the role of Dyrk1A, which is also trisomic in DS and Ts65Dn, and regulates NFAT signaling. They demonstrate that overexpression of both Dyrk1A and Rcan1 in endothelial cells results in greater inhibition of VEGF-mediated endothelial proliferation than in cells overexpressing Rcan1 alone, suggesting that Dyrk1A may be responsible for the increased tumor suppression observed in Ts65Dn/Rcan1^+/+/−^ versus WT.

#### 3.6.3. Synj1

A Synj1 null allele was generated by gene targeting using a vector designed to replace 103 base pairs (bp) from the 3′ portion of the first coding exon, and 1571 bp of the adjacent intron with a neomycin resistance cassette [109]. Mice heterozygous for the null allele are phenotypically normal and fertile. Crosses between heterozygotes produce pups with the expected genotypes, at the expected Mendelian ratio. However, within a few hours, the homozygous null pups become distinguishable from their littermates by the severe reduction in the amount of milk in their stomachs. About 85% of the homozygous null mice die within 24 hours, and the remaining 15% die within 15 days. The latter group exhibit reduced growth, with a 3-fold difference versus littermates at 10 days, and they develop severe weakness, ataxia, and generalized convulsions that can be evoked by the tail flick test. These results clearly indicate that the gene is essential for postnatal development. The absence of Synj1 expression did not alter the expression of a large variety of nerve terminal proteins, including synaptojanin 1 interactors, proteins thought to play a role in synaptic vesicle endocytosis, intrinsic membrane proteins of synaptic vesicles, plasma membrane t-SNAREs, additional proteins thought to play a role in the synaptic vesicle cycle, and enzymes involved in phosphoinositide (PI) metabolism.

The authors demonstrate a 1.6-fold increase of PtdIns(4,5)P_2_ in cultured cortical neurons from null mice versus WT, but no major differences in PtdIns(4)P (they were unable to detect other PI species), and that the increase in PtdIns(4,5)P_2_ is due to a reduction in dephosphorylation of PtdIns(4,5)P_2_ to PtdIns(4)P. Electron microscopy of cultured cortical neurons showed an increased number of clathrin-coated vesicles localized around the synaptic vesicle cluster, and that the great majority are isolated vesicles (separated from the plasma membrane). The PI binding properties of clathrin coat proteins suggest that the increased number of clathrin-coated vesicles is due, at least in part, to increased PtdIns(4,5)P_2_. Similar results were obtained in cell-free assays in which protein-free liposomes from crude brain lipid extracts were incubated in brain cytosol plus ATP and GTP. Cytosol from null animals produced a 4-fold higher number of coated vesicles than WT cytosol. Biochemical analysis showed a larger pool of clathrin and AP-2 bound to liposomes incubated with the null cytosol. This difference was counteracted by addition of purified synaptojanin 1 to the null cytosol.

Electrophysiological analyses of hippocampal slices from 10-day-old animals suggest that basal properties of synaptic transmission are unchanged in null mice, but that regeneration of a releasable pool for synaptic vesicle release is diminished in hippocampal synapses from null animals resulting in a depression of synaptic response. The authors suggest that the absence of synaptojanin 1 may affect actin dynamics as well (PI are important regulators of the actin cytoskeleton), resulting in trapping of clathrin-coated vesicles within an actin matrix.

The expression of Synj1 in Ts65Dn brain is about 40% greater than in controls as measured by quantitative western blot [115]. This overexpression in Ts65Dn results in a 33% increase in the production of phosphatidylinositol monophosphate (PtdInsP) relative to controls in a brain cytosol assay, using NBD-PtdIns(4,5)P_2_, a fluorescently labeled water-soluble substrate. Reducing the copy number of Synj1 to disomy in Ts65Dn results in reduction of PtdInsP production to control levels. Similarly, HPLC analysis with suppressed conductivity detection demonstrates a ~16% decrease in the mass of PtdIns(4,5)P_2_ in Ts65Dn brain relative to controls. The decrease was fully corrected in brain from Ts65Dn mice disomic for Synj1. Finally, in metabolic labeling studies of phospholipids in cortical synaptosomes, they demonstrate a ~30% decrease in the PtdInsP_2_/PtdA ratio in Ts65Dn versus controls. The authors suggest that increased expression of Synj1 may play a role in the learning deficits observed in Ts65Dn mice. As stated above (Section 3.3.3), the authors demonstrate a learning deficit, as evaluated by the Morris Water Maze test, in mice transgenic for either murine Synj1 or human SYNJ1. Unfortunately, they did not evaluate Ts65Dn mice disomic for Synj1, although they do state that performing this experiment is essential [115]. 

### 3.7. Knockout Mice Possibly Related to the Altered Cancer Spectrum in People with DS

#### 3.7.1. ERG

The v-ets erythroblastosis virus E26 oncogene homolog (avian) gene (ERG, aka p55, and erg-3; Erg is the murine homolog) encodes transcriptional regulator ERG, a member of the erythroblast transformation-specific (ETS) family of transcription factors.

ERG is involved in chromosomal rearrangements in myeloid leukemia, in 5 to 10% of cases of Ewing's sarcoma, resulting in fusion of Erg and a member of the Tet subfamily of RNA-binding proteins. ERG is deleted in a subset of acute lymphoblastic leukemias, which may facilitate transformation, and is suggestive of a role for ERG in DS childhood leukemia. Chromosomal rearrangements result in control of ERG expression by the androgen-responsive 5′ elements of TMPRSS2 in more than half of all prostate cancers. Thus, there is strong evidence that ERG has an important role in hematopoiesis, and that it is a potent oncogene. So far, no ERG transgenic mice have been reported.

A germline mutation of Erg, designated Erg^Mld2^, was obtained via a genetic screen for regulators of hematopoietic stem cell function [146]. Direct sequencing revealed that the mutation is a thymidine to cytosine transition in exon 12, causing a substitution of proline for serine at residue 329 in the first *α*-helix of the DNA-binding Ets domain. Pulse chase experiments in a human embryonic kidney cell line indicated the mutant protein has a half-life similar to that of WT Erg, suggesting that it is stable *in vivo*. Electrophoretic mobility-shift assays using radiolabeled DNA and titration with cold competitor DNA show that the mutant retains DNA binding ability and binds the E74 enhancer element (a known Erg-binding site) with an affinity similar to the WT protein. However, reporter assays demonstrate that the mutant's ability to transactivate transcription is negligible, and that it cannot promote megakaryocyte differentiation when expressed in human erythroleukemic cell line K562. 

No Erg^Mld2/Mld2^ mice from matings of mice heterozygous for the Erg^Mld2^ allele were identified at weaning, indicating the homozygous mice were probably dying during embryogenesis. Analysis of embryos from timed matings shows that homozygous mice are viable at day E10.5, some were dead at E11.5, and none were alive at E13.5. Homozygous embryos at day E10.5 exhibit developmental delay, and culture of yolk sacs from these embryos yielded almost no hematopoietic progenitor-derived colonies of any lineage, demonstrating failure of definitive hematopoiesis in the Erg^Mld2/Mld2^ mice. 

Mice heterozygous for the Erg^Mld2^ allele have lower blood platelet numbers than WT, but are not anemic. Histopathology of tissues from adult thymus, spleen, bone marrow, pancreas, lymph nodes, liver, kidney, bladder, small bowel, skin, skeletal muscle, salivary gland, or femur showed no gross abnormalities. Culture of single-cell suspensions of bone marrow and spleen yielded fewer colonies than WT, and the frequency of progenitor cells of all lineage types was about 50% that of control littermates. Colony-forming assays demonstrate that mice heterozygous for the Erg^Mld2^ allele have fewer committed hematopoietic progenitors and multipotent cells, and a smaller population of lineage-negative Sca-1^+^c-kit^+^ (LSK) cells (representing long-term repopulating hematopoietic stem cells and early hematopoietic progenitors).

Ts65Dn mice at 12 months of age exhibit progressive thrombocytosis, megakaryocytosis, and megakaryocytic dysplasia within bone marrow, extra medullary hematopoiesis in spleen with disrupted splenic architecture, and so forth [147]. Breeding the Erg^MLD2^ mutation into the Ts65Dn background to produce mice disomic for ERG results in amelioration of histopathologic myeloproliferative features to WT levels. Interestingly, Ts1Cje mice, although trisomic for Erg, do not develop myeloproliferative disorder, suggesting that Erg is necessary, but not sufficient, for the Ts65Dn myeloproliferative features.

#### 3.7.2. ETS2

An Ets2 allele (ets2db1) was produced by gene targeting using a vector designed to replace all or part of three exons of the gene coding for the Ets2 DNA binding domain with pMC1NeoA [148], resulting in deletion of a critical portion of the gene (a large fusion transcript is observed), and production of a truncated protein that binds to an Ets2 antibody [149]. Mice homozygous for the allele are not obtained from heterozygous matings due to a defect in formation of extraembryonic tissues. They can be obtained via complementation using tetraploid embryos (which produce functional extraembryonic tissues) [150]. 

At birth, ets2db1 homozygous mice exhibit curly whiskers. After ~2 weeks of age, in addition to curly whiskers, the mice exhibit wavy hair, and a slightly rounded forehead. Whole mount analysis of skin shows misalignment of hair follicles, resulting in ingrown curly hairs that fail to penetrate the epidermis. Mice deficient for TGF*α* and mice with a point mutation in the EGF receptor have a similar whisker-hair-hair follicle phenotype. The mice are fertile, and lymphoid and myeloid cell development is not significantly different from WT. 

An Ets2 hypomorphic allele (Ets2A72) was produced by knock in gene targeting, in which the threonine target of Erk phosphorylation is replaced by alanine [151]. Ets2A72 homozygous mice exhibit normal fertility and longevity. They do not develop the hair and hair follicle abnormalities found in rescued Ets2db1 homozygous mice. Histological analysis of 50 organs did not reveal any unusual abnormalities. Mammary gland development in females is normal.

Since Ets2db1 homozygous mice die due to placental insufficiency, Wei et al. [152] employed the Ets2A72 allele to produce an Ets1/Ets2 double “null” to investigate the role of both genes in the ras/Raf/Mek/Erk pathway and prevent rescue of the individual null phenotypes. Mutations in both genes result in abnormal angiogenesis in development, and full lethality by about day E14.5 (Ets2 nulls (db1 and fl) acting essentially the same as mutated Ets2 (A72)). Both genes promote epithelial cell survival in angiogenesis. Both genes are proto-oncogenes and may act in endothelial cells to affect tumor angiogenesis.

Misregulation of ETS2 is associated with cancer, and some studies suggest that increased dosage of ETS2 in DS contributes to a reduced risk of cancer [126]. Ts65Dn mice are trisomic for Ets2. In an elegant series of experiments, Sussan et al. [153] demonstrated that trisomy of Ets2 in Ts65Dn and Ts1Rhr mice suppresses the occurrence of intestinal tumors when these mice are bred with the *Apc* ^*Min*⁡^ mice that have a highly increased incidence of intestinal tumors. These studies are consistent with the proposed role of ETS2 in reducing tumor incidence in DS. As mentioned above, Ts65Dn mice develop cranial skeleton and thymus anomalies. Similar anomalies were seen in transgenic mice that constitutively overexpress a processed Ets2 transcript under metallothionein promoters. To evaluate the role of native Ets2 in the craniofacial and thymus phenotypes of DS, Hill et al. [36] used these mice to show that the reduction in Ets2 expression in these mice does not rescue thymus abnormalities, and mostly does not rescue cranial skeleton abnormalities, except for mesoderm-derived elements (the superoinfero height of the occipital bone is reduced by 16% in Ts65Dn, Ets2+/− versus euploid but is reduced by 4% in Ts65Dn versus euploid). These experiments confirm a role for Ets2 in the suppression of tumors in DS, but Ets2 does not play a major role in skeletal or thymus abnormalities seen in Ts65Dn mice.

#### 3.7.3. RUNX1

A Runx1 null allele was generated by gene targeting using a vector designed to replace the splice acceptor and first 20 bp of the exon encoding the central 52 amino acids of the Runt homology domain (RHD, required for DNA binding) with a hygromycin B cassette [127]. Homologous recombination also introduces stop codons in all three reading frames, ensuring production of a truncated protein.

Mice heterozygous for the null allele are apparently normal, exhibiting no difference in hematocrits, nucleated blood cell counts, white blood cell differentials, or distribution of peripheral blood lymphocyte subsets as analyzed by fluorescence-activated cell sorting analysis (FACS). However, homozygous nulls die during embryogenesis at about day E12.5. Morphological evaluation of E12.5 null embryos shows extensive hemorrhaging within the ventricle of the central nervous system and the vertebral canal, which appears to originate in the ganglia of the cranial nerves, extending into the third and lateral ventricles. Hemorrhaging was also observed in the pericardial space and peritoneal cavity in most null animals. At E11.5, about 87% of null embryos are viable, and are indistinguishable from heterozygous or WT embryos, except for a slight liver pallor. Microscopic examination of null embryo liver at day 11.5 indicates a complete absence of liver-derived hematopoiesis. No erythroid, myeloid, or megakaryocyte cells were identified, and only primitive nucleated erythrocytes were observed in vascular channels and hepatic sinusoids. Runx1 null embryonic stem cells can differentiate into primitive erythroid cells *in vitro*, but no hematopoietic colonies were obtained in cultures from yolk sac or liver from null embryos, demonstrating that Runx1 is essential for liver hematopoiesis.

Hematopoiesis was not well characterized in Ts65Dn mice, except for one report demonstrating decreased proliferation of CD34^+^ cells *in vitro* [154]. Kirsammer et al. [38] investigated hematopoiesis in Ts65Dn mice and demonstrate that they develop highly penetrant progressive myeloproliferative disease characterized by thrombocytosis, mild anemia, extramedullary hematopoiesis, bone marrow fibrosis, and distorted stem and myeloid progenitor compartments, and they note that the phenotype resembles human chronic idiopathic myelofibrosis (the incidence of which increases with age [155]). To elucidate the role, if any, of increased expression of Runx1, they employed a breeding strategy involving the Runx1 null allele [127] to produce Ts65Dn mice disomic for Runx1. They conclude that increased dosage of Runx1 is not required for development of megakaryocytic hyperproliferation, extramedullary hematopoiesis, and reticulin fibrosis observed in Ts65Dn mice.

Carmichael et al. [156] investigated hematopoiesis in Ts1Cje mice which, as discussed above, are trisomic for a smaller region of Mmu16 than that in Ts65Dn mice. Ts1Cje exhibits a hematopoietic phenotype similar to that observed in Ts65Dn mice, except Ts1Cje mice do not show any sign of development of thrombosis or myeloproliferative disease. This suggests strongly that trisomy of one or more genes within the trisomic region unique to Ts65Dn is responsible for development of thrombosis and myeloproliferative disease, while the other hematopoietic abnormalities are largely caused by trisomy of genes in the trisomic region common to both Ts65Dn and Ts1Cje.

## 4. Conclusions

Recent progress in methods for producing genetically altered mice demonstrates that it is now possible, at least in theory, to produce mice trisomic for any gene found on HSA21 or any mouse chromosomal region syntenic to HSA21, and KO mice for any HSA21 syntenic gene(s). Indeed, a large international effort is underway to produce KO mice for all mouse genes and to assess their phenotypes. Moreover, and equally important, it is possible to completely characterize the genetic alterations in the various mouse models, including alterations in gene number, expression, and structure, which is essential for proper interpretation of the consequences of trisomy of particular genes or chromosomal regions. This capability presents an unprecedented opportunity for unraveling the mechanisms of DS pathogenesis and, on the basis of this information, devising rational therapies for alleviation of the deleterious consequences of Trisomy 21.

The analysis of various mouse models to date allows some preliminary conclusions. By far, the most well-characterized mouse model phenotypically is the Ts65Dn mouse. Considerable evidence suggests that Ts65Dn mice exhibit aspects of aging relevant to DS. Interestingly, even though Ts65Dn mice are trisomic for about 60 Mmu17 genes and are disomic for about 16 Mmu16 genes found on HSA21, their phenotype is remarkably similar to that of the Dp(16)1Yey/+ mice that are trisomic for the entire Mmu16 region syntenic to HSA21, and that have no additional trisomic genes. Of course, differences may be revealed as the mice are more thoroughly characterized. This observation does not mean that HSA21 genes found on Mmu10 or Mmu17 are irrelevant for DS. For example, the Mmu10 or 17 regions may ameliorate some of the effects of trisomy of the Mmu16 region. 

In general, analysis of transgenic and KO mice reveals phenotypes consistent with a given gene's known function and in some cases have helped in elucidating its function. Also, when KO mice have been bred with trisomic mice, reducing a gene's copy number from three to two, the observed effects have been consistent with the gene's function as determined by other studies. 

Often, the point has been brought up that the genetic background of the various mouse models is critical, since response to trisomy may differ depending on this parameter. One should keep in mind, however, that people with DS are certainly not inbred, and an effect seen in a noninbred mouse strain or in multiple genetic backgrounds may be more relevant to the human situation than effects observed in inbred models. It is also important to assess phenotypes in more than one mouse model where possible.

## 5. Future Directions

The results from studies of genetically altered mice, coupled with the ability to produce essentially any mouse model, demonstrate that this approach will play a key role in understanding the DS phenotype, as well as phenotypes related to the biology of aging in people with DS. Studies so far make it clear that the genetic alterations in mouse models can be precisely defined with regard to gene content and alterations in gene expression. Results from studies in which dosage of specific genes in segmentally trisomic mice is reduced to disomy via breeding with relevant KO mice suggests that this is a particularly fruitful approach. As KO mice for more genes become available, the pace of these experiments should accelerate. This approach appears to be more successful in revealing a gene's role in DS than the creation of single-gene transgenic mice. However, the production of transgenic mice to assess the effects of trisomy of specific genes, especially genes associated with a particular disorder, or for which a particularly compelling hypothesis suggests they may have a significant effect, may be worthwhile. Also, there may be strong justification for increasing the dosage of syntenic Mmu10 and 17 genes to trisomy in mice trisomic for Mmu16 regions.

The observation that segmental trisomy for Mmu10 and Mmu17 regions syntenic to HSA21 have relatively minor phenotypic effects appears to limit the regions of HSA21 important in DS. However, this interpretation may not yet be warranted. First, it is possible that continuing characterization of the various segmental trisomy mice may reveal phenotypes relevant to DS that are caused or affected by genes located in these regions. Moreover, it may be that trisomy of these regions interacts with trisomy of the Mmu16 syntenic region, affecting the Mmu16 trisomic phenotype. 

Ts65Dn and other segmental trisomy mouse models serve as treatment models for some aspects of DS, and agents showing a beneficial effect in these mice are either in human clinical trials or will be soon on the basis of their success in ameliorating the symptoms in these models. This brings up an interesting and critical question, namely, is the approach of using particular mouse models valid in preclinical studies, especially when the mechanism of action of a given agent is poorly understood? One could argue that if a particular agent improves a deleterious phenotype reminiscent of those seen in people with DS, this should be sufficient justification for proceeding with human trials. This perspective is problematic in some ways. For example, it is possible that genes on the HSA21 syntenic Mmu10 or 17 regions will influence the result of drug treatment. For example, individuals with DS show an increased sensitivity to cytosine arabinoside and this may help explain the high event-free survival rates seen in treatment of people with DS who have developed AML. This increased sensitivity is attributed, at least partially, to trisomy of the CBS gene, located on Mmu17 in mice. Similarly, individuals with DS show a significantly increased sensitivity to methotrexate, one of the most widely used anticancer drugs, and this may be due to trisomy for the Slc19a1 (reduced folate carrier) gene, which is on Mmu10 [15]. It would be interesting to determine whether making Ts65Dn mice trisomic for CBS or for RFC would increase their sensitivity to cytosine arabinoside or methotrexate, respectively. The appropriate transgenic mice already exist and have been partially characterized. In general, the more one knows about the mechanism of drug's action, the more effectively one may be able to test it in appropriate mouse models. Therefore, mechanistic studies of the effects of possible therapies using mouse models would be extremely worthwhile. 

It is reasonable to expect that therapies to improve intellectual and other disabilities associated with DS and/or aging will soon become available. Mouse models of DS will have played a critical role in this development, and it is virtually certain that they will continue to do so. Thus, mouse models will have made a major contribution to the lives of individuals with DS and their families. Moreover, any feature seen in individuals with DS is also seen in the population without DS. Therefore, use of these mouse models will likely have beneficial effects far beyond the population with DS.
